# Meta-analysis on the association between pathologic complete response and triple-negative breast cancer after neoadjuvant chemotherapy

**DOI:** 10.1186/1477-7819-12-95

**Published:** 2014-04-15

**Authors:** Kunpeng Wu, Qiaozhu Yang, Yi Liu, Aibing Wu, Zhixiong Yang

**Affiliations:** 1 Cancer Center, Affiliated Hospital of Guangdong Medical College, 57 Renmin Road, Zhanjiang, PR China; 2 Cancer Center, Heyuan People’s hospital, Heyuan, PR China; 3 Department of Gynecology and Obstetrics, Women and Child Care Institute of Heyuan, Heyuan, PR China; 4 Guangdong Key Laboratory for Research and Development of Natural Drugs, Guangdong Medical College, Zhanjiang, PR China

**Keywords:** breast cancer, neoadjuvant chemotherapy, pathologic complete response, meta-analysis

## Abstract

**Background:**

Triple-negative breast cancer (TNBC) is a special subtype of breast cancer that is characterized by poor prognosis, strong tumor invasion and a high pathologic complete response (pCR) to neoadjuvant chemotherapy (NAC). The pCR rate is a prognostic factor for TNBC. We aimed to evaluate the relationship between pCR and TNBC after NAC and originally tried to identify factors related to achieving pCR for TNBC using a meta-analysis.

**Methods:**

We systematically searched the literature for pCR and breast cancer after NAC and carefully identified eligibility criteria. The association between pCR and breast cancer subtypes was estimated using Review Manager, while pCR rates for TNBC and non-TNBC were determined using Meta-Analyst.

**Results:**

This analysis included a total of 9,460 cases from 27 studies. The summary odds ratio estimating the relationship between pCR and breast cancer subtypes (TNBC vs non-TNBC) was 3.02 (95% confidence interval (CI), 2.66 to 3.42). The TNBC pCR rate was 28.9% (95% CI, 27.0 to 30.8%) and the non-TNBC was 12.5% (95% CI, 11.7 to 13.4%). From subgroup analyses, we identified the factors associated with the highest pCR rates for TNBC.

**Conclusions:**

TNBC has a higher pCR rate than non-TNBC. In the NAC setting, these factors of platinum-containing, more than six cycles, four kinds of drugs, 16 weeks’ treatment duration and sequential chemotherapy may contribute to increasing the pCR rate.

## Background

Triple-negative breast cancer (TNBC) is a subtype of breast cancer that accounts for approximately 15% of all breast cancers [1,2]. TNBC lacks the three important therapeutic markers for clinical regimens of patients with breast cancer: estrogen receptor (ER), progesterone receptor (PR) and human epidermal growth factor receptor 2 (HER2). Due to the absence of a therapeutic target (endocrine therapy targets the ER and PR, and trastuzumab targets HER2), the prognosis of patients with TNBC is poorer than that of patients with other types of breast cancer. Patients with TNBC are characterized by early recurrence [3,4] and a significantly shorter survival compared with those with non-TNBCs [5,6].

Neoadjuvant chemotherapy (NAC) is increasingly being used in the treatment of large operable breast cancers or to prevent lymph node metastases, where it is as effective as adjuvant chemotherapy and considered a standard of treatment for patients with locally advanced breast cancer [7]. The advantages of NAC in operable breast cancer include: increasing the rate of success of breast-conserving surgery by downstaging the primary tumor load, early prevention of cancer metastasis in lymphonodi or viscera and providing suggestions for selecting the adjuvant chemotherapy regimen through estimating the clinical response to NAC and avoiding a potentially ineffective treatment in adjuvant chemotherapy.

Interestingly, several clinical studies on NAC for breast cancers have shown that TNBC has lower survival and higher relapse rates among all breast cancer subsets but has a higher rate of pathologic complete response (pCR) to NAC than other phenotypes and patients with pCR have excellent survival [1,8]. In other words, patients with TNBC who do not have pCR are at increased risk of early relapse and death [1,9]. pCR has been proven to be a prognostic factor for breast cancer by von Minckwitz and Xiangnan Kong [10,11]. Consequently, pCR plays a very significant role in predicting prognosis and clinical management for patients with TNBC.

We therefore performed a meta-analysis aiming to report the association between NAC and pCR for TNBC. It was also our purpose to observe which factors are potentially related with pCR in TNBC treated with NAC, such as NAC cycles, drugs and schedules.

## Methods

### Literature search

The MEDLINE, EMBASE and Cochrane Library databases were systematically searched to September 2013. Publications with the following search words in the title, abstract or key words were included: breast cancer, TNBC, NAC, preoperative chemotherapy, pathologic complete response, pathologic complete remission and pathologic response. The studies identified through the search were independently screened by two authors (KW and AW) for inclusion. Any disagreements were arbitrated by a third author (ZY). We did not limit our search by language, country, race or date.

### Inclusion and exclusion criteria

Studies performed using humans regardless of sample size were included if they met the following criteria: papers studying the association between NAC and pCR in TNBCs; all cases definitely diagnosed as breast cancer and where distant metastasis was excluded; ER, PR, HER2 measured by immunohistochemistry (IHC) and/or fluorescence *in situ* hybridization of primary cancer tissue; pCR explicitly defined; and detailed statistics had to be reported (i.e. patient numbers and percentage of pCR). Any investigations that did not meet all inclusion criteria and cross-sectional studies were excluded. If data were duplicated in more than one paper, the most recent paper was included in the analysis.

### Data extraction

Data were independently extracted by two authors (QY and YL) using the same standardized table. The fields extracted included first author, year of publication, NAC schedule (type, number of cycles, interval and treatment duration), and number and percentage of patients achieving pCR in TNBC and non-TNBC. For articles with the same population resources or overlapping datasets, data were extracted and reported as a single trial.

### Statistical analysis

The Cochrane Collaboration Review Manager 5.1 and Meta-Analyst Beta 3.13 statistical software were used for this meta-analysis. The *χ*^2^ and *I*^2^ test methods were used to evaluate the heterogeneity of the odds ratios (ORs) in the studies. When *I*^2^ < 50% and *P* > 0.05 for *χ*^2^, indicating heterogeneity in the results, the heterogeneity in the studies was considered acceptable and the fixed-effect model with the Mantel–Haenszel method was used for the two-arm meta-analysis or the inverse variance method was used for the single-arm meta-analysis. Otherwise, a random-effect model with the DerSimonian and Laird method was adapted for both the one- and two-arm meta-analyses. Each study was weighted according to the sample size.

Subgroup analyses were executed for NAC cycles, drugs and schedules. The sensitivity was analyzed by excluding small cases studies (defined as <100 cases) and changing the effect model to estimate confidence. Potential publication bias was evaluated using funnel plots. An asymmetric plot indicates there was potential publication bias; otherwise, the plot should be shaped like a funnel.

### Ethical standards

This study complies with the current laws of China.

## Results

### Eligible studies

**Figure 1:**
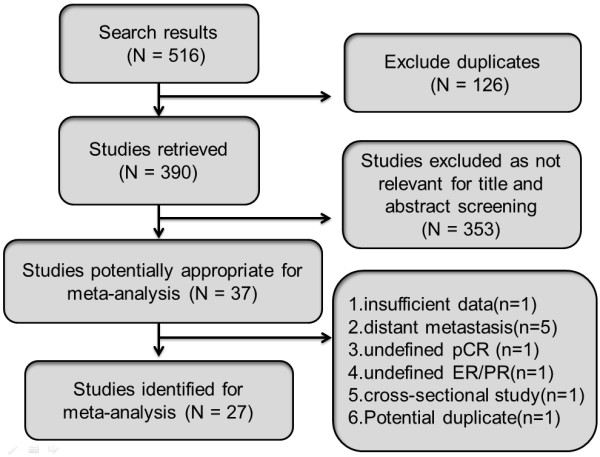
**Flow chart used to identify relevant literature.** ER, estrogen receptor; pCR, pathologic complete response; PR, progesterone receptor.

We identified 516 studies in the three databases and their bibliographies of relevant clinical trials. After excluding duplicates (*n* = 126), the titles and abstracts of all remaining studies (*n* = 390) were reviewed. Of these 390 studies, we excluded 353 that did not meet the selection criteria. After reviewing the full text of the remaining 37 studies, we ultimately included 27 studies [1,8,9,12-35] in the final analysis. Ten studies were excluded from the final review for these reasons: insufficient data (*n* = 1) [36], cross-sectional study (*n* = 1) [37], distant metastasis (*n* = 5) [38-42], undefined pCR (*n* = 1) [43] or undefined hormone receptor (*n* = 1) [44]. The same populations were reviewed in two papers [23,45], the data were extracted and reported as a single study. Figure 1 shows a flow diagram with the numbers of relevant studies.

### Study characteristics

In all, 27 studies published between 2005 and 2012 were included in this meta-analysis. Table 1 shows the main characteristics of all of the studies. A total of 9,460 cases from the 27 studies that had pathological results and clinical data were included. Enrollment of participants across the studies was from 1985 to 2009. Most studies enrolled patients who had been diagnosed with breast cancer stages II and III (*n* = 17), but some studies also recruited stage I patients (*n* = 8) and a few studies recruited non-metastatic patients (*n* = 3). According to these studies, the percentage of patients achieving pCR after NAC was 3.1 to 66.7% (TNBC, 14.6 to 66.7%; non-TNBC, 3.1 to 39.0%). NAC consisted of an anthracycline and/or taxane with other chemotherapeutic regimens.

**Table 1:** Characteristics of the eligible studies

**Author**	**Year**	**Tumor stage**	**Neoadjuvant chemotherapy**	**TNBC**		**pCR rate of TNBC**	**non-TNBC**		**pCR rate of non-TNBC**	**OR (95% CI)**
				**pCR**	**no-pCR**		**pCR**	**no-pCR**		
Rouzier R [8]	2005	I, II, III	12 weeks of P followed by FAC 4 courses (weekly P (80 mg/m^2^) × 12 + FAC × 4 or 3 weekly P (225 mg/m^2^) × 4 + FAC × 4)	10	12	45.50%	11	49	18.30%	3.71 (1.28, 10.76)
Carey LA [1]	2007	II, III	A 60 mg/m^2^ + C 600 mg/m^2^ every 2 weeks or 3 weeks for 4 cycles, either alone or as the first component of a sequential AC-taxane neoadjuvant regimen	9	25	26.50%	8	69	10.40%	3.10 (1.08, 8.93)
Goldstein NS [12]	2007	IIA to IIIC	FAC every 3 weeks × 6 FEC every 3 weeks × 6 AC every 2 weeks (dose dense) × 4 then paclitaxel every 2 weeks (dose dense) × 4 AC every 3 weeks × 4 then P every 1 week × 4	12	9	57.10%	16	31	34.00%	2.58 (0.90, 7.41)
Keam B [13]	2007	II, III	D (75 mg/m^2^ or 60 mg/m^2^) and A (60 mg/m^2^ or 50 mg/m^2^) by intravenous infusion every 3 weeks for 3 cycles	8	39	17.00%	3	95	3.10%	6.50 (1.64, 25.78)
Liedtke C [9]	2008	I, II, III	FAC; FEC; weekly or once every 3 weeks P/D followed by FAC; weekly or once every 3 weeks P/D followed by FEC	57	198	22.40%	98	765	11.40%	2.25 (1.56, 3.23)
Bidard FC [14]	2008	I, II, III	FEC (F 500 mg/m^2^, E 100 mg/m^2^, C 500 mg/m^2^) or FAC (F 500 mg/m^2^, A 60 mg/m^2^, C 500 mg/m^2^), every 3 weeks for 4 to 6 cycles	21	99	17.50%	7	166	4.00%	5.03 (2.06, 12.26)
Julka PK [15]	2008	IIA to IIIB	4 cycles (21 days) of Gem 1,200 mg/m^2^ + A 60 mg/m^2^, 4 cycles of Gem 1,000 mg/m^2^ plus Cis 70 mg/m^2^	7	7	50.00%	6	16	27.30%	2.67 (0.65, 10.88)
Sánchez-Muñoz A [16]	2008	II, III	Schedule A: E 90 mg/m^2^ + C 600 mg/m^2^ d1 for 3 cycles followed by a second sequence with P 150 mg/m^2^ + Gem 2,500 mg/m^2^ d1 ± trastuzumab 2 mg/kg/week according to HER2 status Schedule B: A 40 mg/m^2^ d1 + P 150 mg/m^2^ + Gem (2,000 mg/m^2^) d2, 2 weekly for 6 cycles	14	10	58.30%	17	58	22.70%	4.78 (1.80, 12.66)
Sirohi B [17]	2008	M0	F 200 mg/m^2^ daily with E 60 mg/m^2^ and Cis 60 mg/m^2^ both repeating 3 weekly for 6 courses	1	5	16.70%	5	49	9.30%	1.96 (0.19, 20.26)
Darb-Esfahani S [18]	2009	T2 to 3, N0 to 2, M0	A 50 mg/m^2^ + D 75 mg/m^2^ every 14 days for 4 cycles or 4 cycles A 60 mg/m^2^ plus C 600 mg/m^2^ every 21 days followed by D 100 mg/m^2^ every 21 days for 4 cycles	8	25	24.20%	5	78	6.00%	4.99 (1.50, 16.65)
Sikov WM [19]	2009	IIA to IIIB	Cb (AUG = 6) every 4 weeks and P 80 mg/m^2^ weekly for 16 weeks, and weekly trastuzumab was added for HER2(+) status	8	4	66.70%	16	25	39.00%	3.13 (0.81, 12.11)
Bhargava R [20]	2010	I, II, III	Anthracycline-based therapy: AC, FEC; taxane-based therapy: T/P + Cb. In many cases a sequential combination of anthracycline and taxane was given: AC-T. The total number of cycles ranged from 4 to 10 with an average of 6	24	55	30.40%	24	256	8.60%	4.65 (2.46, 8.80)
Chang HR [21]	2010	II, III	D (75 mg/m^2^) and Cb (AUC = 6) were administered every 3 weeks for 4 cycles. Patients with HER2(+) tumors were randomized to receive either additional weekly trastuzumab preoperatively or TC alone	6	5	54.50%	13	47	21.70%	4.34 (1.14, 16.51)
Chavez-Macgregor M [22]	2010	M0	Taxane administered: P 175 to 250 mg/m^2^ on d1, 3 weekly for 4 cycles; P 80 mg/m^2^ weekly for 12 doses; or D 100 mg/m^2^ on d1, 3 weekly for 4 cycles Anthracycline regimens (3 to 6 cycles): F 500 mg/m^2^, E 100 mg/m^2^ and C 500 mg/m^2^ on d1, d3 weekly; F 500 mg/m^2^ on d1, d4, E 75 mg/m^2^ and C 500 mg/m^2^ on d1, 3 weekly	95	395	19.40%	165	1419	10.40%	2.07 (1.57, 2.73)
Chen XS [23]	2010	T3 to 4, any N, M0; any T, N2 to 3, M0	VE: V 25 mg/m^2^ d1, d8 + E 60 mg/m^2^ d1, d3 weekly; PCb: P 80 mg/m^2^ + Cb AUC = 2 d1, d8, d15, 4 weekly; CEF: C 500 mg/m^2^, E 75 mg/m^2^ and F 500 mg/m^2^ d1, 3 weekly; CTF: C 500 mg/m^2^, THP 50 mg/m^2^, and F 500 mg/m^2^ d1, 3 weekly; CEF➝T: D 75 mg/m^2^ d1, 3 weekly; ED: E 60 mg/m^2^ + D 75 mg/m^2^, 3 weekly	9	44	17.00%	19	153	11.00%	2.07 (0.86, 4.96)
Huober J [24]	2010	I, II, III	6 to 8 cycles of TAC (D 75 mg/m^2^, A 50 mg/m^2^, C 500 mg/m^2^ on d1, every 3 weeks) or 2 cycles of TAC followed by four cycles of V 25 mg/m2 on d1, d8 + capecitabine 1,000 mg/m^2^ orally twice a day on d1 to d14 every 3 weeks	198	311	38.90%	147	820	15.20%	3.55 (2.77, 4.56)
Kim SI [25]	2010	M0	A (50 mg/m^2^, d1) + D (75 mg/m^2^, d1) chemotherapy (AT) every 3 weeks for 3 cycles	16	60	21.10%	10	181	5.20%	4.83 (2.08, 11.21)
Pierga JY [26]	2010	II, III	E (75 mg/m^2^) + C (750 mg/m^2^) intravenously every 3 weeks for 4 cycles followed by D (100 mg/m^2^) every 3 weeks for 4 cycles with or without trastuzumab (8 mg/kg at first infusion then 6 mg/kg) every 3 weeks	23	55	29.50%	14	57	19.70%	1.70 (0.80, 3.64)
Straver ME [27]	2011	T1 to 3, N0 to 2, M0	AC (6 cycles of A 60 mg/m^2^ and C 600 mg/m^2^ every 3 weeks) or AD (6 cycles of A 50 mg/m^2^ and D 75 mg/m^2^, every 3 weeks)	16	41	28.10%	13	181	6.70%	5.43 (2.43, 12.17)
Bernsdorf M [28]	2011	T2 to 3, N0 to 3b, M0	4 cycles of EC (E 90 mg/m^2^ and C 600 mg/m^2^) plus 12 weeks of daily treatment with gefitinib 250 mg or EC plus 12 weeks’ treatment with placebo. Chemotherapy was administered every 3 weeks	12	70	14.60%	1	47	2.10%	8.06 (1.01, 64.06)
Iwata H [29]	2011	T1c to 3, N0, M0; T1 to 3, N1, M0	4 cycles of D (75 mg/m^2^) administered intravenously every 21 days followed by 4 cycles of FEC (F 500 mg/m^2^, E 100 mg/m^2^ and C 500 mg/m^2^) administered intravenously on d1 every 21 days before surgery	14	15	48.30%	16	84	16.00%	4.90 (1.99, 12.09)
Loo CE [30]	2011	T2 to 4, N1 to 3, M0	Either ER(+) or (–), received 6 courses of AC, administered in a dose-dense schedule (every 2 weeks). A minority received 6 courses of capecitabine + D or doxorubicin + D	16	41	28.10%	22	119	15.60%	2.11 (1.01, 4.40)
Medioni J [31]	2011	II, III	Six 2-weekly courses of Gem 1,000 mg/m^2^ + D 75 mg/m^2^ on d1, d15 and V 25 mg/m^2^ + E 100 mg/m^2^ on d29, d43. Patients with an objective response on d56 then received another cycle of Gem + D on d57 and V + E on d71	9	13	40.90%	7	43	14.00%	4.25 (1.32, 13.65)
Nakahara H [32]	2011	T1 to 4	HER2(–) tumors started with CE (E 75 mg/m2 × d1 + C 100 mg × daily for 14 days with 7 days’ rest) for 4 or 6 cycles. HER2(+) tumors initiated with CE (E 90 mg/m^2^ × d1 or E 50 mg/m^2^ × d1, d8 and C 100 mg × daily for 14 days with 7 days’ rest)	5	13	27.80%	3	65	4.40%	8.33 (1.77, 39.27)
Wu J [33]	2011	II, III	P (175 mg/m^2^) or D (75 mg/m^2^) + doxorubicin (60 mg/m^2^) or E (90 mg/m^2^) every 21 days for a total of 4 cycles	14	40	25.90%	24	171	12.30%	2.49 (1.19, 5.25)
Le Tourneau C [34]	2012	II, III	4 cycles of intensified FAC (A 70 mg/m^2^ d1, C 700 mg/m^2^ d1 + d8, and F 700 mg/m^2^ d1 to d5) every 3 weeks	9	10	47.40%	3	30	9.10%	9.00 (2.03, 39.93)
Ono M [35]	2012	II, III	Anthracycline-based regimen (AC: A 60 mg/m^2^ + C 600 mg/m^2^ or CEF: C 600 mg/m^2^ + E 100 mg/m^2^ + F 600 mg/m^2^) Taxane-based regimen (weekly P 80 mg/m^2^ or triweekly D 75 mg/m^2^) Anthracycline and taxane sequentially or concurrently (A 50 mg/m^2^ + D 60 mg/m^2^, AC or CEF followed by weekly P or triweekly D)	26	66	28.30%	9	70	11.40%	3.06 (1.34, 7.02)

A, adriamycin; C, cyclophosphamide; Cb, carboplatin; CI, confidence interval; Cis, cisplatin; d, day; D, docetaxel; E, epirubicin; F, 5-fluorouracil; Gem, gemcitabine; HER2, human epidermal growth factor receptor 2; OR, odds ratio; P, paclitaxel; pCR, pathologic complete response; THP, pirarubicin; TNBC, triple-negative breast cancer; V, vinorelbine; T, Taxane/Taxotere; AUC, area under the curve.

### Pathologic complete response and breast cancer subtypes (triple-negative breast cancer and non-triple-negative breast cancer)

**Figure 2:**
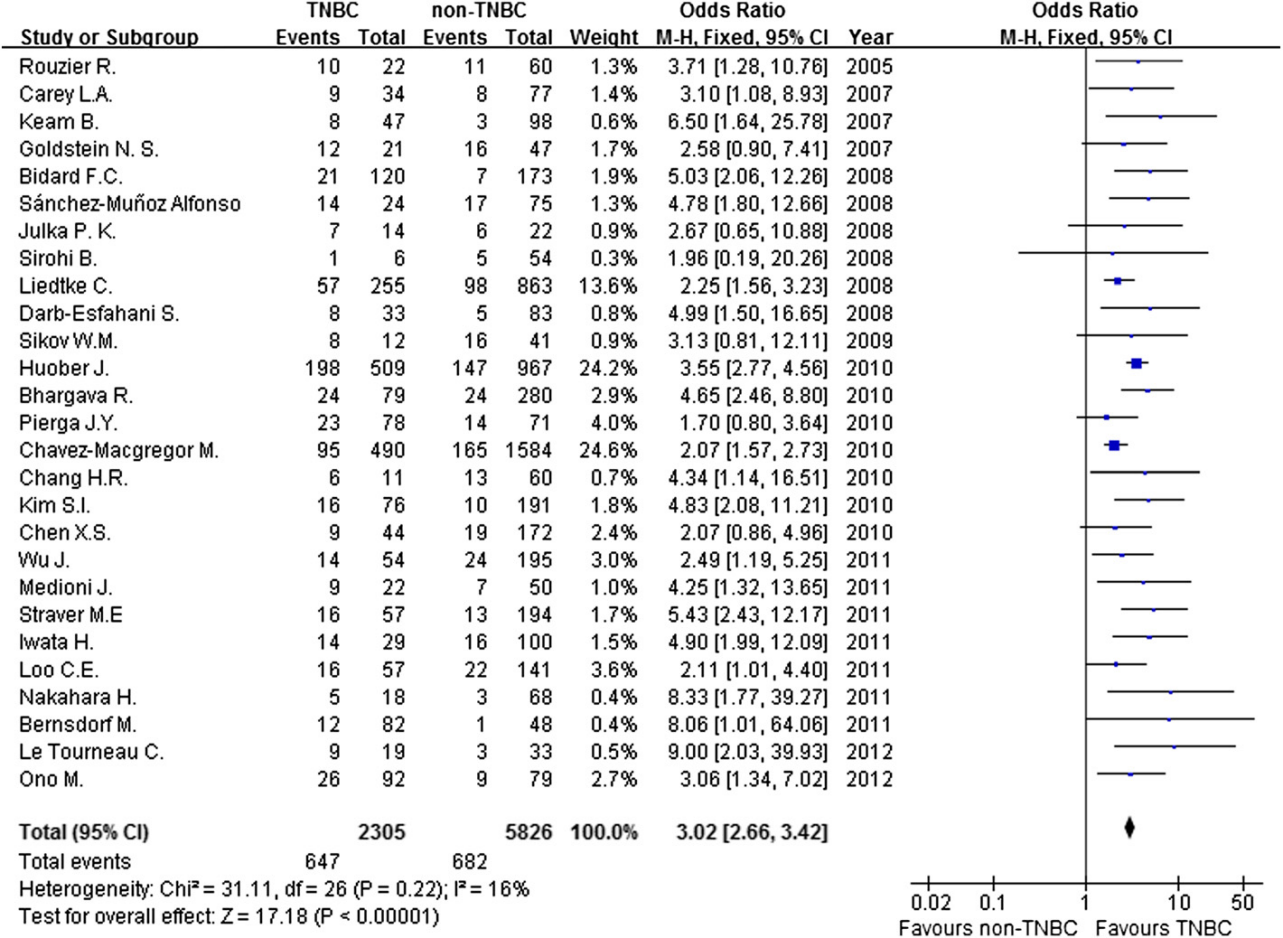
**Forest plot of odds ratio for achieving pCR after NAC between TNBC and non-TNBC.** CI, confidence interval; TNBC, triple-negative breast cancer; M-H, Mantel–Haenszel method.

**Figure 3:**
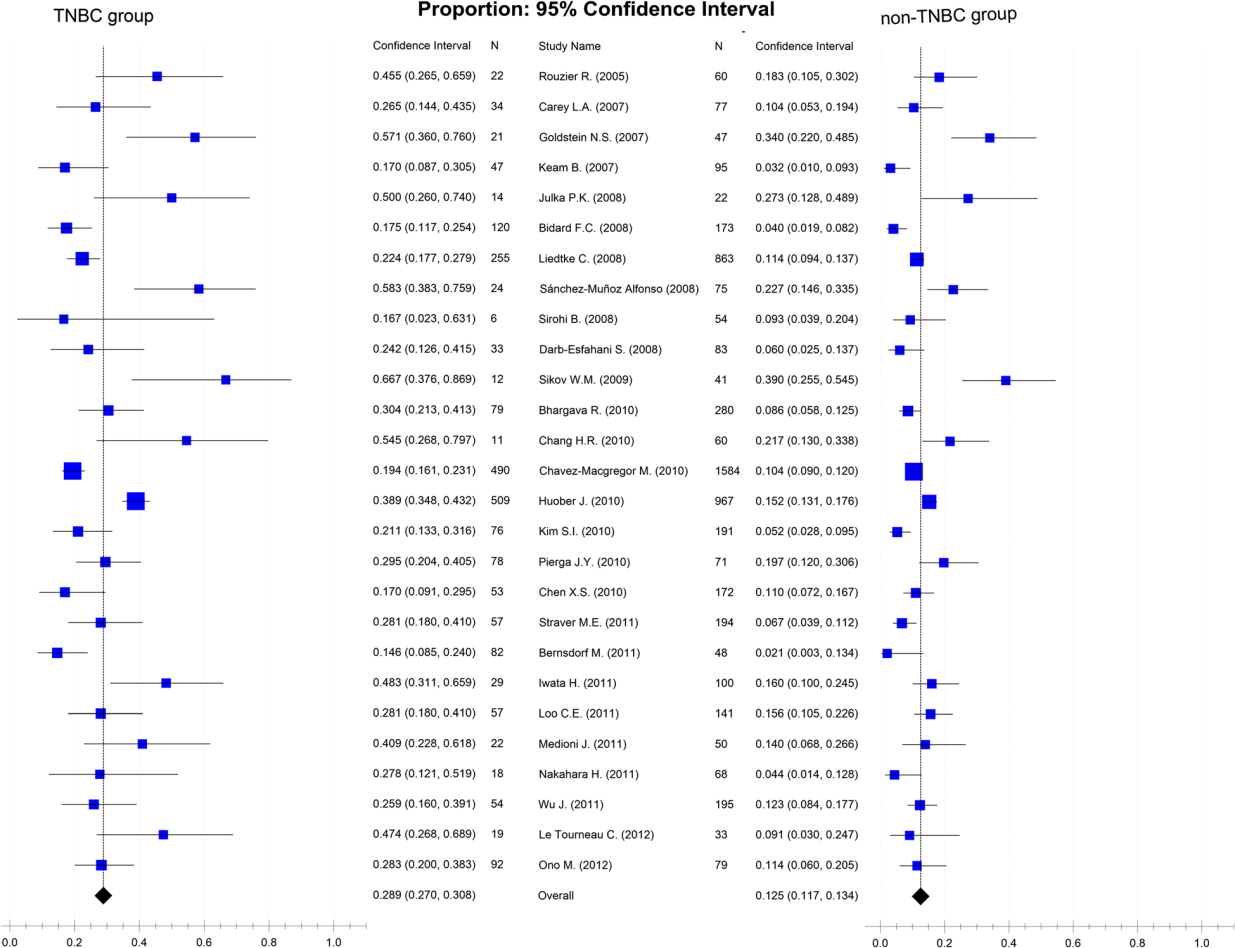
**Forest plots of pooled percentage of achieving pCR after NAC for TNBC and non-TNBC groups.** TNBC, triple-negative breast cancer.

Figure 2 shows the association between pCR and breast cancer subtypes (TNBC and non-TNBC) after NAC. In a fixed-effects meta-analysis of all 27 studies, TNBC has a better pCR rate than non-TNBC (the overall summary estimate OR was 3.02; 95% CI, 2.66 to 3.42) with no obvious evidence of heterogeneity (*I*^2^ = 16%, *P* = 0.22). Figure 3 summarizes the percentage of patients achieving pCR after NAC in TNBC and non-TNBC groups. In a single-group fixed-effects meta-analysis of all 27 studies, the overall summary estimated pCR rate was 28.9% (95% CI, 27.0 to 30.8%) in TNBC and 12.5% (95% CI, 11.7 to 13.4%) in non-TNBC. There was no obvious evidence of heterogeneity (*I*^2^ = 44.1% and *I*^2^ = 43.8%, respectively).

The subgroup analysis outcomes are shown in Table 2. The initially planned subgroup of chemotherapy intermission was not used due to a lack of similar data in these studies. Instead, we used subgroups for NAC treatment duration, which was defined as the period of time that patients were treated with NAC. These subgroup analyses involve treatment cycle (<4 cycles, 4 cycles, 6 cycles or >6 cycles), types of chemotherapy regimen (anthracycline-based, taxane-containing, platinum-containing, gemcitabine-containing), the number of chemotherapy drugs (two kinds of drugs, three kinds of drugs or four kinds of drugs), treatment duration (<12 weeks, 12 weeks, 16 weeks or >16 weeks) and chemotherapy schedule (conventional vs sequential chemotherapy). We discovered that the pCR rate was higher with TNBC than with non-TNBC for all subgroups. A single-group meta-analysis of all 27 studies [1,8,9,12-35] identified the subgroups (four kinds of chemotherapy drugs, >6 cycles, platinum-containing chemotherapy, 16 weeks’ treatment duration, sequential chemotherapy) with the highest pCR rate for both TNBC and non-TNBC patients.

**Table 2:** Subgroup analyses of various factors related to achieving pathologic complete response

**Category**	**Number of studies (references)**	**Summary estimate odds ratio (95% CI)**	**Heterogeneity, *I***^**2 **^**(%)**	**pCR rate (95% CI)**			
				**TNBC (%)**	**Heterogeneity, *I***^**2 **^**(%)**	**non-TNBC (%)**	**Heterogeneity, *I***^**2 **^**(%)**
**Cycles of NAC**							
<4 cycles	2 [13,25]	5.27 (2.57, 10.79)	0	19.6 (13.5, 27.6)	0	4.6 (2.7, 7.8)	0
4 cycles	6 [15,21,28,33,34,45]	3.51 (2.21, 5.57)	0	29.2 (23.0, 26.3)	41.4	15.0 (11.8, 18.8)	35.1
6 cycles	3 [17,27,30]	3.10 (1.84, 5.22)	35	27.6 (20.3, 36.3)	0	11.0 (8.2, 14.7)	41.3
>6 cycles	3 [8,26,29]	2.77 (1.66, 4.61)	41	36.8 (28.8, 45.6)	33.8	17.8 (13.4, 23.3)	0
**Types of NAC regimen**							
Anthracycline-based	19 [1,8,9,12-14,16-18,25-34]	3.19 (2.63, 3.88)	7	26.8 (24.1, 29.6)	39.8	12.1 (10.8, 13.5)	43.2
Taxane-containing	10 [8,13,18,19,21,25,26,29],[33,45]	3.29 (2.41, 4.48)	0	30.5 (25.9, 35.5)	38.2	14.9 (12.6, 17.5)	44.8
Platinum-containing	4 [17,19,21,45]	3.10 (1.59, 6.03)	0	44.2 (30.8, 58.5)	31.1	21.3 (16.3, 27.3)	43.6
Gemcitabine-containing	2 [15,31]	3.49 (1.42, 8.57)	0	44.5 (29.3, 60.8)	0	18.8 (11.2, 29.8)	30.2
**The number of drug in NAC**							
Two kinds of drugs	9 [13,15,19,21,25,27,32,33],[45]	3.89 (2.75, 5.49)	0	28.7 (23.8, 34.2)	36.3	12.9 (10.7, 15.5)	46.1
Three kinds of drugs	4 [14,17,26,34]	2.39 (1.77, 3.23)	16	22.5 (18.8, 26.5)	19.8	11.2 (9.5, 13.3)	43.6
Four kinds of drugs	4 [8,9,29,31]	4.83 (2.80, 8.35)	0	45.7 (35.8, 55.9)	0	15.5 (11.4, 20.6)	0
**Total treatment duration of NAC**							
<12 weeks	2 [13,25]	5.27 (2.57, 10.79)	0	19.6 (13.5, 27.6)	35.4	4.6 (2.7, 7.8)	0
12 weeks	7 [14,15,21,28,30,33,34]	3.42 (2.34, 4.99)	0	24.9 (20.5, 29.9)	42	13.3 (10.7, 16.3)	42.6
16 weeks	2 [19,45]	2.88 (1.27, 6.56)	0	44.2 (28.4, 61.3)	41.4	24.8 (17.7, 33.7)	46.7
>16 weeks	6 [8,17,24,26,27,29]	3.47 (2.80, 4.30)	7	37.6 (34.0, 41.2)	25.8	14.7 (12.9, 16.6)	38
**NAC schedules**							
Conventional chemotherapy	8 [13-15,17,21,25,27,45]	4.40 (3.02, 6.42)	0	24.1 (19.8, 29.0)	35.6	9.7 (7.7, 12.1)	44.5
Sequential chemotherapy	4 [8,26,29,31]	2.96 (1.85, 4.72)	20	37.4 (30.0, 45.5)	22.5	17.2 (13.2, 22.1)	0

CI, confidence interval; NAC, neoadjuvant chemotherapy; pCR, pathologic complete response; TNBC, triple-negative breast cancer.

**Figure 4:**
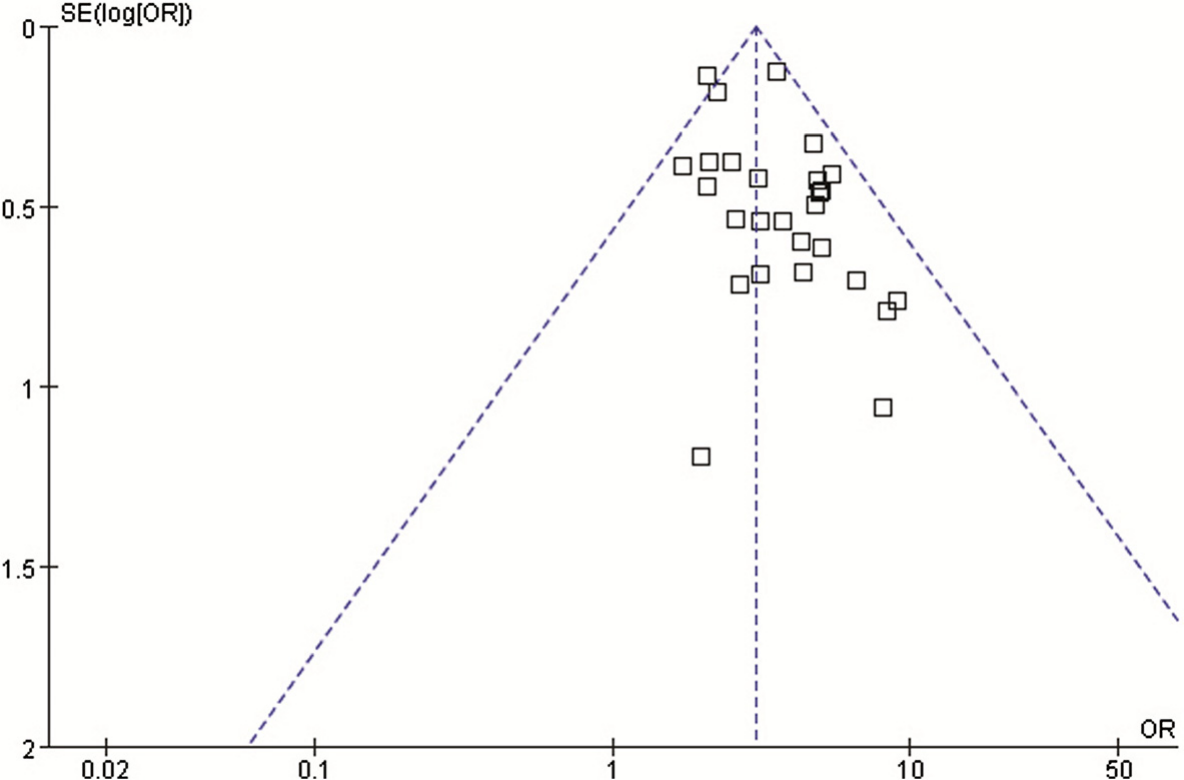
**Funnel plot for identifying publication bias in the relationship for achieving pCR between TNBC and non-TNBC.** OR, odds ratio.

**Figure 5:**
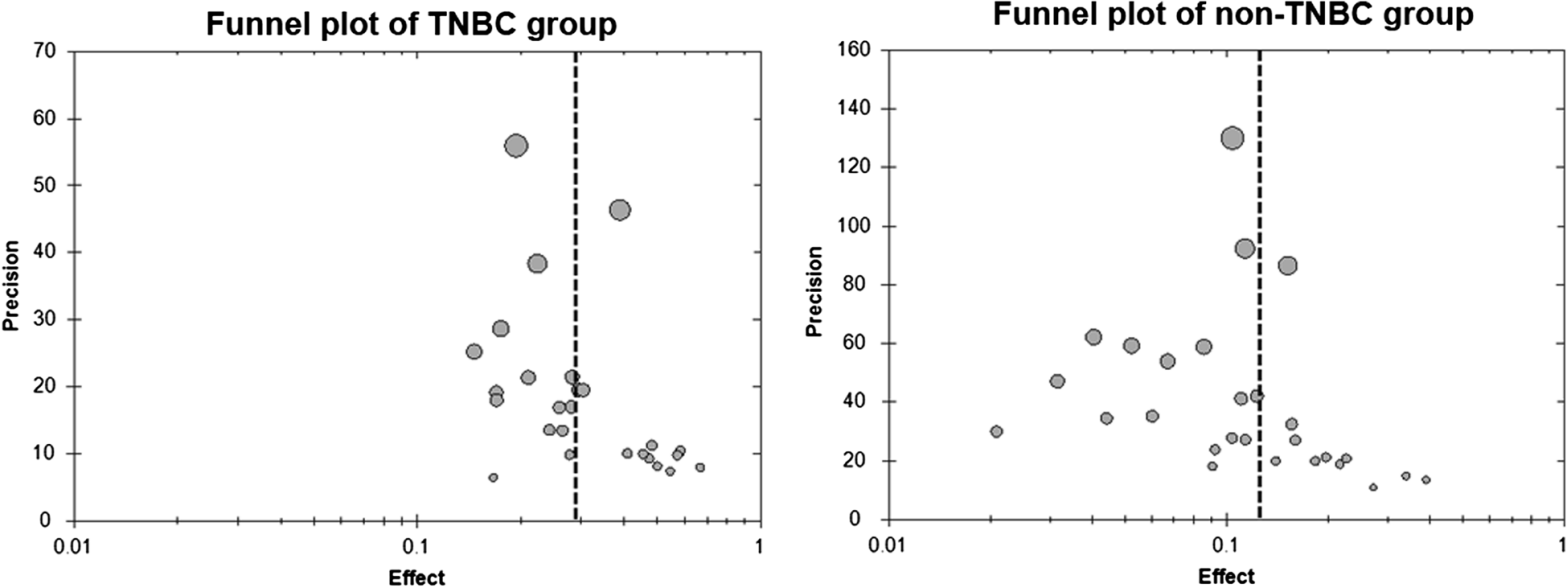
**Funnel plot for identifying publication bias for the pooled pCR rates in TNBC and non-TNBC.** TNBC, triple-negative breast cancer.

A sensitivity analysis shown that excluding small cases studies and changing the effect model had little effect on estimated OR and pCR rate and did not change the strength of the association between NAC and pCR for TNBC and non-TNBC. The ORs were 3.13 (95% CI, 2.66 to 3.68) for excluding small cases studies and 2.92 (95% CI, 2.56 to 3.34) for changing the effect model. For TNBC patients, the odds of pCR were 27.2% (95% CI, 25.3 to 29.2%) for excluding small cases studies and 30.5% (95% CI, 25.9 to 35.5%) for changing the effect model. For non-TNBC patients, the odds of pCR were 11.5% (95% CI, 10.7 to 12.5%) for excluding small case studies and 12.5% (95% CI, 10.4 to 14.9%) for changing the effect model. Funnel plots were generated to test for potential publication bias (Figures 4 and 5). Potential publication biases were found in these funnel plots.

## Discussion

TNBC is a subtype of breast cancer that has particular biological features such as high pathologic grade, poor prognosis, short survival, strong tumor invasion, and a high incidence of local relapse and distant metastasis [46]. In addition, a high pCR rate after NAC is also a significant characteristic of TNBC, and pCR has been proved to be a typical marker predictive of clinical response and survival in TNBC patients [11,47]; however, diverse pCR rates have been reported in various studies. In this meta-analysis of 27 studies containing 9,460 cases, pCR rates were 28.9% (95% CI, 27.0 to 30.8%) for 2,952 cases of TNBC and 12.5% (95% CI, 11.7 to 13.4%) for 6,508 cases of non-TNBC. Patients with TNBC have a higher probability of achieving pCR than those with non-TNBC (OR, 3.02; 95% CI, 2.66 to 3.42); that is, the TNBC pCR rate is about two times that of non-TNBC and TNBC exhibits a better response to NAC than non-TNBC.

With the rapid development of molecular and genetic diagnosis techniques, the heterogeneity of breast cancer has been discovered. Based on the analysis of RNA expression profiles, four distinct molecular subtypes of breast cancer (luminal subgroup, basal-like subgroup, HER2 subgroup and normal-like breast tumors) were identified and reported by Perou *et al*. [48]. The basal-like breast cancer is IHC characterized by overexpression of cytokeratin 5/6/14 and epidermal growth factor receptor and lack of expression of ER, PR and HER2 [49,50]. There is intrinsic homology but incomplete overlap between IHC-defined TNBC and molecular-defined basal-like breast cancer. Nearly 80% of TNBC cases have a basal-like molecular profile [51,52]. In addition to the basal-like profile, TNBC encompasses other molecular subtypes, particularly normal-like and claudin-low [53]. In this meta-analysis, we found four studies of participants with basal-like breast cancer and an estimated pCR rate of 42.5% (95% CI, 32.4 to 53.2%). There was no obvious evidence of heterogeneity (*I*^2^ = 31.2%). Basal-like breast cancer has a higher pCR rate than TNBC. Thus, there is evidence that the subtype of triple-negative cancers is heterogeneous and we cannot simply consider them a single group.

Both anthracyclines and taxanes are usually used in the neoadjuvant treatment of breast cancer, and patients respond well to them. Of the 27 studies in this meta-analysis, 19 used anthracycline-based NAC and 11 used taxane-containing regimens. The pCR rates for TNBC were 26.8% (95% CI, 24.1 to 29.6%) for the anthracycline-based group and 30.5% (95% CI, 25.9 to 35.5%) for the taxane-containing group, a non-significant difference. Interestingly, the platinum-containing group had a higher pCR rate than either the anthracycline-based or taxane-containing groups. It is believed that most TNBC cells are expected to have a *BRCA1* mutation or absence [54,55], which is useful for the treatment of TNBC since loss of *BRCA1* function in TNBC is related to the sensitivity of DNA-damaging chemotherapy agents (platinum, alkylating agents, etc.) and may also be related to the resistance of spindle poisons (taxanes and vinblastines) [56]. TNBC is strongly related to germ-line mutations in the *BRCA1* gene, and 90% of *BRCA1*-mutated cancers are TNBC [57]. Some researchers have demonstrated that the addition of platinum agents to anthracycline and/or taxane regimens in NAC has promise for outcomes [58]. Although the gemcitabine-containing group included two studies with 108 cases [15,31], we should not ignore this group, which achieved the highest pCR rate. Due to lack of sufficient cases to support gemcitabine use in NAC for TNBC, more clinical trials should be implemented.

A hypothesis-generating study indicated that TNBC/basal-like breast cancer had a poorer response to anthracycline-based therapy compared with other breast cancer subtypes [59]. The results of this study were laterally validated through this meta-analysis, which indicated that the anthracycline-based group had the lowest pCR. Although some new drugs have been used in NAC for TNBC (such as EGFR inhibitors (NCT00491816), epothilones (NCT01097642) and ixabepilone (NCT01097642)), the platinum-containing strategy was still the first choice in most clinical trials of TNBC and NAC (NCT00887575, NCT01194869 and NCT00813956). It is a pity that the final reports of these clinical trials have not been submitted; however, these reports were very valuable for providing informative references for the clinical practice. Based on the platinum-containing subgroup analysis of 292 cases from 4 studies [17,19,21,45] and some cell biology research [54-57], we recommend the platinum-containing strategy should be used in NAC for TNBC.

From the subgroup analyses of cycles, drug types, treatment duration and chemotherapy schedules (Table 2), we observed that groups of more than six cycles, four kinds of drugs, 16 weeks treatment duration and sequential chemotherapy obtained the highest pCR rate in the respective subgroups for TNBC (36.8%: 95% CI, 28.8 to 45.6%; 45.7%: 95% CI, 35.8 to 55.9%; 37.6%: 95% CI, 34.0 to 41.2%; 37.4%: 95% CI, 30.0 to 45.5%, respectively). We found that the NAC scheme of FAC/TEC-T or T-FAC/TEC had greater weight in the subgroups for four kinds of drugs [8,9,29] and sequential chemotherapy for TNBC [8,26,29] (the weight was 76.6% and 84.5%, respectively). This chemotherapy scheme may be a good choice of NAC for TNBC.

Three meta-analyses were published recently on breast cancer and pCR. Von Minckwitz *et al*. [11] presented a meta-analysis of 6,377 operable and non-metastatic breast cancer patients, who received neoadjuvant anthracyclines or taxanes. They discerned various definitions of pCR and evaluated the prognostic impact of pCR on disease-free survival and overall survival in various breast cancer subgroups. The authors concluded that pCR should be conservatively defined as ypT0 ypN0 excluding ductal carcinoma *in situ* and that pCR is an effective mark of survival for TNBC, luminal B and non-luminal (*HER2*-positive). Kong *et al*. [10] completed a meta-analysis that included 16 studies with 3,776 patients with breast cancer to determine whether pathologic response after NAC predicts outcomes. The authors concluded that the pathologic response is prognostic for relapse-free survival, disease-free survival and overall survival. Houssami *et al*. [60] reported a meta-analysis with two analysis models to provide evidence of the association between various factors for breast cancer and the rates of achieving pCR. Our meta-analysis included 27 studies with 9,460 non-metastatic breast cancer patients, and we aimed to evaluate the association between pCR and breast cancer subtypes (TNBC and non-TNBC) after NAC, and originally tried to identify factors related to achieving pCR for TNBC.

There are some potential limitations in this meta-analysis. Hormone receptor assessment varies across different studies, and different IHC standards are used to define positivity. Most studies define ER/PR-negative IHC using the threshold of <10% immunoreactive cells. The American Society of Clinical Oncology and the College of American Pathologists guidelines for IHC dictate that a threshold of <1% of cells should be used to define ER/PR-negative so that more patients with breast cancer will receive endocrine therapy [53,61]. Moreover, it is unfortunate that sufficient detailed survival data for performing survival analysis are lacking.

## Conclusions

In summary, this meta-analysis provides strong evidence that TNBC has a higher pCR rate than non-TNBC. In the NAC setting, these factors of platinum-containing, more than six cycles, four kinds of drugs, 16 weeks’ treatment duration and sequential chemotherapy may result in a higher pCR rate. This information provides valuable direction for clinicians performing relevant clinical studies in the future.

## Abbreviations

CI: confidence interval; ER: estrogen receptor; HER2: human epidermal growth factor receptor 2; IHC: immunohistochemistry; NAC: neoadjuvant chemotherapy; OR: odds ratio; pCR: pathologic complete response; PR: progesterone receptor; TNBC: triple-negative breast cancer.

## Competing interests

No potential competing interests were disclosed.

## Authors’ contributions

KW and QY performed statistical analysis and wrote the manuscript; KW, YL, AW and QY performed literature search and stratified the data; AW and ZY provided meaningful discussion key points; KW and AW revised and edited the manuscript. All authors read and approved the final manuscript.
